# Lomax tangent generalized family of distributions: Characteristics, simulations, and applications on hydrological-strength data

**DOI:** 10.1016/j.heliyon.2024.e32011

**Published:** 2024-05-31

**Authors:** Sajid Mehboob Zaidi, Zafar Mahmood, Mintodê Nicodème Atchadé, Yusra A. Tashkandy, M.E. Bakr, Ehab M. Almetwally, Eslam Hussam, Ahmed M. Gemeay, Anoop Kumar

**Affiliations:** a Department of Statistics, Govt. Graduate College B.R., Bahawalpur, Pakistan; b Government SE Graduate college Bahawalpur, Bahawalpur, Pakistan; c National Higher School of Mathematics Genius and Modelization, National University of Sciences, Technologies, Engineering and Mathematics, Abomey, Republic of Benin; d Department of Statistics and Operations Research, College of Science, King Saud University, P.O. Box 2455, Riyadh 11451, Saudi Arabia; e Faculty of Business Administration, Delta University for Science and Technology, Gamasa 11152, Egypt; f Helwan University, Department of Mathematics, Faculty of Science, Cairo, Egypt; g Department of Mathematics, Faculty of Science, Tanta University, Tanta 31527, Egypt; h Department of Statistics, Faculty of Basic Science, Central University of Haryana, Mahendergarh 123031, India

**Keywords:** Lomax distribution, Central, reliability and generating functions, Moments and its derivatives, Linear Representation, Simulation study, Bias, Mean square error

## Abstract

This article proposes and discusses a novel approach for generating trigonometric G-families using hybrid generalizers of distributions. The proposed generalizer is constructed by utilizing the tangent trigonometric function and distribution function of base model G(x). The newly proposed family of uni-variate continuous distributions is named the “Lomax Tangent Generalized Family of Distributions (LT-G)” and structural-mathematical-statistical properties are derived. Some special and sub-models of the proposed family are also presented.

A Weibull-based model, ‘The Lomax Tangent Weibull (LT-W) Distribution,” is discussed and the plots of density (pdf) and hazard (hrf) functions are also explained. Model parameter estimates are estimated by employing the maximum likelihood estimation (MLE) procedure. The accuracy of the MLEs is evaluated through Monte Carlo simulation. Last but not least, to demonstrate the flexibility and potential of the proposed distribution, two actual hydrological and strength data sets are analyzed. The obtained results are compared with well-known, competitive, and related existing distributions.

## 1 Introduction and motivations of study

Several sophisticated families/classes of probability distributions have been developed by utilizing well-known classical continuous distributions, offering statistical compositions more application freedom see Table 1. The G-Family is motivated by the need for a flexible distribution with unique properties. A literature survey highlights established distributions, emphasizing the growing interest in families that offer versatility. The transformer (X) family within the G-Family distribution is explored, detailing its transformative role. This subfamily's distinctive features and applications are discussed, showcasing its contribution to addressing specific challenges. Existing studies leveraging the G-Family, particularly the transformer (X) family, are reviewed, underscoring its utility in diverse fields. For modeling data in a diverse range of application domains, including actuarial sciences, industry, Biological and earth sciences, the aforementioned new families have worn out see Table 1. Financial, life-cycle analysis and insurance applications need these distributions to be extended or generalized. These generalized distributions offer more flexibility by supplementing the underlying model with extra parameters (location, scale, inequality, and shape).

**Table 1:** Recent generalized families and adopted approach or technique.

S. No.	Developer(s)	Produced G-Family	Adopted Approach
1	Almetwally [10]	Odd Weibull Inverse TL distribution	Odd Generator
2	Hassan [30]	Kumaraswamy Inverted TL distribution	Generalization
3	Almetwally [11]	Marshall–Olkin Alpha Power-X distribution	APT
4	Almongy [12]	Extended Odd Weibull Rayleigh	Extension
5	Al-Babtain [3]	Flexible Burr XG family	Generalization
6	Hamdani [29]	Type-I Quasi Lambert Family	Lambert Function
7	Altun [14]	Additive Odd-G Family	Additive Model Structure
8	Aljarrah [7]	Generators of Distributions	Quantile Function
9	Salahuddin [51]	New Family of Distributions	GOF and Flexibility
10	Korkmaz [37]	New Class of Distributions	Hjorth's IDB Generator
11	Cordeiro [24]	XGamma Family	Parameter Induction
12	Korkmaz [35]	TL Gen. Odd Log-Logistic Family	Odd Generator
13	Alzaatreh [15]	G-Classes of Distributions	T-X Methodology
14	Bhatti [16]	On Burr III MO Family	Hazard Rate Function
15	Alizadeh [6]	Another Odd LL Logarithmic-G	Odd Generator
16	Kooks [36]	New G-Classes of Distributions	Extended Model

The groundbreaking work of [15], which established and described the *transformed (T) - transformer (X) family*, marked the beginning of an abrupt shift in generalized classes. The proposed distribution function (cdf) is:

$$F(x)=\int_{a}^{W[G(x)]}\;r(t)\;dt.$$

Table 1 shows several recent noteworthy generalized families of distributions, along with their goals or methodologies: In this concern, the first motivation stems from statisticians’ need to create unique and adaptable models with distinctive mathematical and graphical properties. The second is that basic and non-generalized models produce a poorer fit than extended and generalized models, particularly in real-world circumstances. The third crucial aspect is that the data behave more complexly than is often anticipated across various fields. The fourth is to introduce a variety of families and models. The fifth is the mixture/hybrid generalizers based on algebraic and trigonometric function combinations are still to be created and investigated.

During this research, it is observed that (1) – incorporating trigonometric functions into already-existing algebraic families and distributions opens up new research directions for mathematicians and statisticians. (2) – The trigonometric functions are helpful in many ways like reducing the parameter(s), and simple in-series expansions. (3) – These functions additionally improve the flexibility properties of the baseline cumulative distribution function (cdf) G(x) while maintaining relative balance and simplicity in their formulations. This, in our opinion, is a positive development for contemporary statistics that broadens the field's understanding of trigonometric-G families and improves the body of statistical literature.

Table 2 presents the existing research on trigonometric-based families and distributions, which inspired and pushed us to write this paper. Furthermore, the following essential elements serve as the study's pillars: (1) – Many non-trigonometric G-families have been contributed to the statistical literature. In contrast, trigonometric ones have been ignored in favor of algebraic generalizers. (2) – In response to the trend in modeling data, researchers have developed more efficient and effective statistical models based on trigonometric functions. (3) – Mixed generalizers for algebraic and trigonometric functions have yet to be developed and implemented.

**Table 2:** Generalized classes using trigonometric functions.

No.	Developer(s)	Methodology adopted	Presented Trigonometric Model
1	Raab-Green [47]	Cosine function	Cosine Distribution
2	Nadarajah-Kotz [44]	Trig. functions	Beta Trigonometric Distribution
3	Chakraborty [17]	Sine in skewed model	Sin-skew Logistic Distribution
4	Souza [56]	Trig. transformation	New Trig. Classes of Prob. Distributions
5	Kharazmi [34]	Hyperbolic Sine function	Hyperbolic Sine-Weibull Distribution
6	Chesneau [22]	Sine-cosine mutation	Cosine Sine (CS) Distribution
7	Mahmood [40]	New sine generalizer	A New Sine-G Family of Distributions
8	Chesneau [21]	Sine in survival function	Sine Kumaraswamy-G Family
9	Babtain [1]	Sine in unit interval class	Sine Topp-Leone-G Family
10	Ali [5]	Sine function	On Sine Power Lomax Model
11	Nagarjuna [46]	Properties only	The Sine Power Lomax Model
12	Shrahili [53]	Estimation only	Estimation of Sine Inverse Exponential Model
13	Shrahili [54]	Sine in inverse model	Sine Half-logistic Inverse Rayleigh Distribution
14	Gang Shi [52]	Sine in uncertainty	Sine Entropy of Uncertain Random Variables
15	Kharazmi [33]	Tan in actuarial science	Arctangent -X family of distributions
16	Muse [43]	Tan transformation	Tan log-logistic distribution

Trigonometric generalized families of statistical distributions are a class of probability distributions that arise in the context of circular data analysis. These distributions are used to model data that are measured on a circle, such as directions, angles, or phases.

The basic idea behind trigonometric generalized families of distributions is to use trigonometric functions to define the density function of the distribution. The most commonly used trigonometric functions are the sine and cosine functions, although other functions can also be used. There are several families of trigonometric generalized distributions, including Circular uniform distribution, von Mises distribution, Wrapped normal distribution, Bingham distribution, and Kent distribution. Trigonometric generalized families of distributions have wide applications in various fields, including biology, geology, ecology, psychology, and engineering, among others.

Overall, trigonometric generalized statistical distributions provide a mathematical framework to describe, analyze, and model data with periodic behavior. They find applications in diverse fields, ranging from physics and engineering to finance and biology, enabling researchers and practitioners to gain insights, make predictions, and solve problems related to cyclic phenomena.

In this article, the mixture/hybrid generalizers developed disbursing algebraic and trigonometric functions (Tangent) are created and investigated. The proposed generalizer is embedded in the existing Lomax model to introduce a new G-family (LT-G) of distributions employing the T-X methodology of generating families of distributions. The proposed family is a new work in the field of generalized classes of distributions. During this research, it is observed that (i) – The incorporation of trigonometric functions into already-existing classical, algebraic families and distributions opens up new research directions for mathematicians and statisticians. (ii) – The trigonometric functions are helpful and simple, reduce overparameterization, and much more. (iii) – These functions additionally improve the flexibility properties of the underlying cumulative distribution function (cdf) G(x) while maintaining relative balance and simplicity in their formulations. In our opinion, this is a positive development for contemporary statistics that broadens the field's understanding of trigonometric-G families and improves the body of statistical literature. (iv) – The interest in the modeling of directional and proportional data motivated the applied researchers to develop and utilize the trigonometric functions-based models which can more efficiently and potentially handle these data sets. For more reading about reliability, subject see [19], [20], [39], [59].

The format of the paper is as follows: A brief introduction to generalization and parameter induction is given in the first Section. The following section examines the Lomax Tangent-G (LT-G) family. Section three contains models generated exclusively by LT-G. The LT-G family's mathematical characteristics are inferred in the fourth Section. Parameter estimation and a simulation study are covered in Section 5. The data analysis is found in section six. In Section 8, there are some closing remarks.

## 2 Lomax tangent generalized (LT-G) family of distributions

This section outlines the planned family's construction process and the important functions of the new family.

### 2.1 Development methodology

Consider a r.v. “T” such that 0<t<∞ possessing density $r(t)=\frac{c}{k}\;{\left[1+(\frac{t}{s})\right]}^{-k-1}$. The introduced trigonometric generalizer is $W[G(x)]=\left(\tan\left(\frac{\pi }{2}\;G(x)\right)\right)$ fulfilling all requirements of T-X generalization procedure. Then, the main functions of LT-G family are:

(1) $$F(x)=\int_{0}^{\left(\tan(\frac{\pi \;G(x)}{2})\right)}\;r(t)\;dt=1-{\left[1+\left(\frac{\tan\left(\frac{\pi }{2}\;G(x)\right)}{s}\right)\right]}^{-k},$$

(2) $$f(x)=\frac{\pi \;k}{2\;s}\;g(x)\;\left[\sec^{2}\;\left(\frac{\pi }{2}\;G(x)\right)\right]\;{\left[1+\left[\left(\frac{\tan\left(\frac{\pi }{2}\;G(x)\right)}{s}\right)\right]\right]}^{-k-1}.$$

## 3 Unique LT-G generated models

The LT-G family of distributions' unique models generated using (1) or (2) are shown here:

### 3.1 Lomax tangent Weibull (LT-W) distribution

The LT-W distribution's cdf, pdf, and hrf, in sequence, are:

(3) $$F(x)=1-{\left[1+\left(\frac{\tan\left(\frac{\pi }{2}\;(1-e^{-{\left(\frac{x}{a}\right)}^{b}})\right)}{s}\right)\right]}^{-k},$$

$$f(x)=\frac{\pi \;k}{2\;s}\;\frac{b}{a}\;{\left(\frac{x}{a}\right)}^{b-1}\;e^{-{\left(\frac{x}{a}\right)}^{b}}\;\left[\sec^{2}\;\left(\frac{\pi }{2}\;(1-e^{-{\left(\frac{x}{a}\right)}^{b}})\right)\right]\times {\left[1+\left[\left(\frac{\tan\left(\frac{\pi }{2}\;(1-e^{-{\left(\frac{x}{a}\right)}^{b}})\right)}{s}\right)\right]\right]}^{-k-1},$$

$$h(x)=\frac{\pi \;k}{2\;s}\;\frac{b}{a}\;{\left(\frac{x}{a}\right)}^{b-1}\;e^{-{\left(\frac{x}{a}\right)}^{b}}\;\left[\sec^{2}\;\left(\frac{\pi }{2}\;(1-e^{-{\left(\frac{x}{a}\right)}^{b}})\right)\right]\times {\left[1+\left[\left(\frac{\tan\left(\frac{\pi }{2}\;(1-e^{-{\left(\frac{x}{a}\right)}^{b}})\right)}{s}\right)\right]\right]}^{-1}.$$

**Figure 1:**
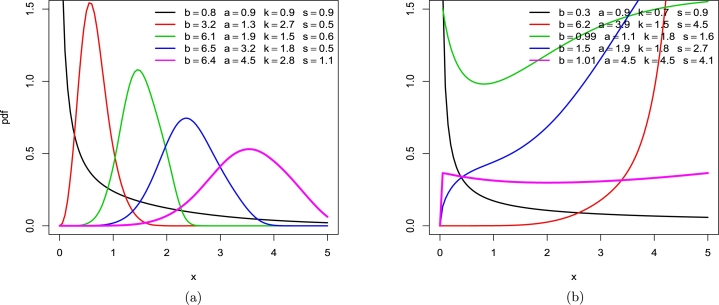
LT-W's plots (a) density (b) hazard rate function on specified parametric values.

The density and hazard rate of the LT-W distribution are depicted in the following graphs for various parametric parameters. The LT-W distribution's pdf is shown in Fig. 1 (a), and its hazard rate is shown in Fig. 1 (b).

### 3.2 Lomax tangent gamma (LT-Ga) distribution

The LT-Ga distribution's cdf, pdf and hrf, in sequence, are:

$$F(x)=1-{\left[1+\left(\frac{\tan\left(\frac{\pi }{2}\;\left(\frac{\gamma (x,\beta )}{\Gamma (\beta )}\right)\right)}{s}\right)\right]}^{-k},$$

$$f(x)=\frac{\pi \;k}{2\;s}\;\frac{1}{\Gamma (\beta )}\;x^{\beta -1}\;e^{-x}\;\left[\sec^{2}\;\left(\frac{\pi }{2}\;\left(\frac{\gamma (x,\beta )}{\Gamma (\beta )}\right)\right)\right]\;{\left[1+\left(\frac{\tan\left(\frac{\pi }{2}\;\left(\frac{\gamma (x,\beta )}{\Gamma (\beta )}\right)\right)}{s}\right)\right]}^{-k-1},$$

$$h(x)=\frac{\pi \;k}{2\;s}\;\frac{1}{\Gamma (\beta )}\;x^{\beta -1}\;e^{-x}\;\left[\sec^{2}\;\left(\frac{\pi }{2}\;\left(\frac{\gamma (x,\beta )}{\Gamma (\beta )}\right)\right)\right]\;{\left[1+\left(\frac{\tan\left(\frac{\pi }{2}\;\left(\frac{\gamma (x,\beta )}{\Gamma (\beta )}\right)\right)}{s}\right)\right]}^{-1}.$$

**Figure 2:**
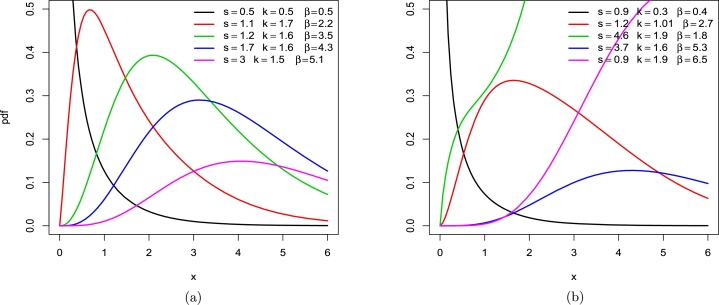
LT-Ga's plots (a) density (b) hazard rate function on specified parametric values.

The density and hazard rate of the LT-Ga distribution are depicted in the following graphs for various parametric parameters. The LT-Ga distribution's pdf is shown in Fig. 2 (a), and its hazard rate is shown in Fig. 2 (b).

### 3.3 Lomax tangent Rayleigh (LT-R) distribution

The LT-R distribution's cdf, pdf, and hrf, in sequence, are:

$$F(x)=1-{\left[1+\left(\frac{\tan\left(\frac{\pi }{2}\;\left(1-e^{-\beta \;X^{2}}\right)\right)}{s}\right)\right]}^{-k},$$

$$f(x)=\frac{\pi \;k}{2\;s}\;2\;\beta \;x\;e^{-\beta \;x^{2}}\;\left[\sec^{2}\;\left(\frac{\pi }{2}\;\left(1-e^{-\beta \;X^{2}}\right)\right)\right]\;{\left[1+\left[\left(\frac{\tan\left(\frac{\pi }{2}\;\left(1-e^{-\beta \;X^{2}}\right)\right)}{s}\right)\right]\right]}^{-k-1},$$

$$h(x)=\frac{\pi \;k}{2\;s}\;2\;\beta \;x\;e^{-\beta \;x^{2}}\;\left[\sec^{2}\;\left(\frac{\pi }{2}\;\left(1-e^{-\beta \;X^{2}}\right)\right)\right]\;{\left[1+\left[\left(\frac{\tan\left(\frac{\pi }{2}\;\left(1-e^{-\beta \;X^{2}}\right)\right)}{s}\right)\right]\right]}^{-1}.$$

**Figure 3:**
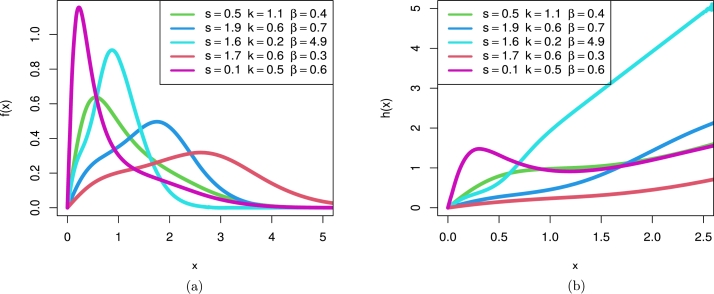
LT-R's plots (a) density (b) hazard rate function on specified parametric values.

Here are the plots of the LT-R distribution's density and hazard rate function for various parameter values. LT-R density is bimodal, as shown in Fig. 3 (a) while Fig. 3 (b) shows LT-R hazard rate exhibited shapes.

### 3.4 Lomax tangent exponential (LT-E) distribution

The LT-E distribution's cdf, pdf, and hrf, in sequence, are:

$$F(x)=1-{\left[1+\left(\frac{\tan\left(\frac{\pi }{2}\;(1-e^{-\beta \;x})\right)}{s}\right)\right]}^{-k},$$

$$f(x)=\frac{\pi \;k}{2\;s}\;\beta \;e^{-\beta \;x}\;\left[\sec^{2}\;\left(\frac{\pi }{2}\;\left(1-e^{-\beta \;x}\right)\right)\right]\;{\left[1+\left[\left(\frac{\tan\left(\frac{\pi }{2}\;\left(1-e^{-\beta \;x}\right)\right)}{s}\right)\right]\right]}^{-k-1},$$

$$h(x)=\frac{\pi \;k}{2\;s}\;\beta \;e^{-\beta \;x}\;\left[\sec^{2}\;\left(\frac{\pi }{2}\;\left(1-e^{-\beta \;x}\right)\right)\right]\;{\left[1+\left[\left(\frac{\tan\left(\frac{\pi }{2}\;\left(1-e^{-\beta \;x}\right)\right)}{s}\right)\right]\right]}^{-1}.$$

**Figure 4:**
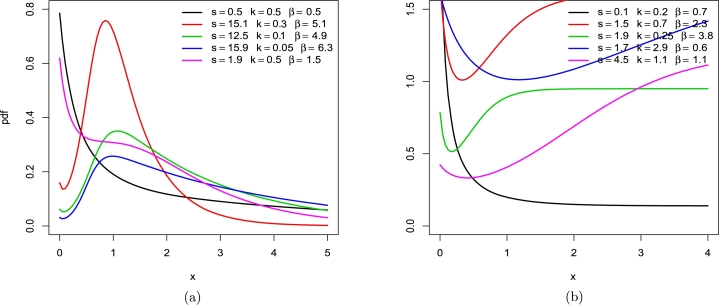
LT-E's plots (a) density (b) hazard rate function on specified parametric values.

Here are the plots of the LT-E distribution's density and hazard rate function for various parameter values. LT-E density shapes are shown in Fig. 4 (a) while Fig. 4 (b) shows LT-E hazard rate exhibited shapes.

## 4 Mathematical properties

A few well-known mathematical features are derived in this part of LT-G distribution.

### 4.1 Quantile function

The quantile function (qf) which is obtained directly inverting by (3) in this way

(4) $$Q(u)=G^{-1}\left[\frac{2}{\pi }\;\tan^{-1}\left[s[{\left(1-u\right)}^{\frac{-1}{k}}-1]\right]\right].$$

Applications of Eq. (4) are given by:

• Replacement any standard model, by using equation (4) to simulate the pdf, histogram, exact cdfs for these data can be accomplished.
• Eq. (4) can be passed down to take median=Q(1/2), measures of kurtosis and skewness.

### 4.2 Effective series expansions

The compilation of CDF in power series is a very useful intellectual process. A suggestion is presented here to demonstrate that the proposed cdf accepts the planned expansion.

$$F(x)\;=\;\sum_{i=1}^{\infty }\;\sum_{j=0}^{\infty }\;\left[-\left(\begin{array}{c} -k\\ i \end{array}\right)\right]\;s^{-i}\;b_{j}(i)\;{\left[\frac{\pi }{2}\right]}^{\left(2j+i\right)}\;{\left[G(x)\right]}^{\left(2j+i\right)}.$$

Then there's

$$F(x)\;=\;\sum_{i=1}^{\infty }\;\sum_{j=0}^{\infty }\;W_{i,j}\;H_{\left(2j+i\right)}(x),$$

where

$$W_{i,j}=s^{-i}\;b_{j}(i)\;{\left(\frac{\pi }{2}\right)}^{\left(2j+i\right)},$$

MATHEMATICA supports the following power series.

$${\left[\tan\left(\frac{\pi G(x)}{2}\right)\right]}^{i}=\sum_{j=0}^{\infty }\;b_{j}(i)\;{\left(\frac{\pi G(x)}{2}\right)}^{\left(2j+i\right)}.$$

Also $b_{0}(i)=1,b_{1}(i)=\frac{i}{3},b_{2}(i)=\frac{i(5\;i+7)}{90}$, etc. and $H_{\left(2j+i\right)}(x)={\left[G(x)\right]}^{\left(2j+i\right)}$ represents the exp-G cdf with the power parameter (2j+i). As a result, *X* density may be described as a combination of exp-G densities of infinite summation; thus,

(5) $$f(x)=\;\sum_{i=1}^{\infty }\;\sum_{j=0}^{\infty }\;w_{i,j}\;h_{\left(2j+i\right)}(x),$$

such that $h_{\left(2j+i\right)}(x)$ represents the exp-G density function with the power parameter (2j+i). Following that, a random variable with the density function $h_{\left(2j+i\right)}(x)$ is marked by $Y_{\left(2j+i\right)}∼\text{exp-G}(2j+i)$.

### 4.3 Density function's shapes

The mathematical forms of the density function may be determined, and the roots can be found by solving:

(6) $$\frac{d\;\log\;f(x)}{dx}=\frac{g^{\prime }(x)}{g(x)}+\pi \;g(x)\;\tan\left(\frac{\pi }{2}\;G(x)\right)+\frac{\pi \;(-k-1)g(x)\sec^{2}\left(\frac{1}{2}\pi G(x)\right)}{2s\left(\frac{\tan\left(\frac{1}{2}\pi G(x)\right)}{s}+1\right)},$$

and there may be several roots to (6). Let $\lambda (x)=d^{2}\;\log[f(x)]/d\;x^{2}$. As we know

$$\lambda (x)=\frac{g^{\prime \prime }(x)}{g(x)}-\frac{g^{\prime }{\left(x\right)}^{2}}{g{\left(x\right)}^{2}}+\frac{\pi (-k-1)g^{\prime }(x)\sec^{2}\left(\frac{1}{2}\pi G(x)\right)}{2s\left(\frac{\tan\left(\frac{1}{2}\pi G(x)\right)}{s}+1\right)}+\pi g^{\prime }(x)\tan\left(\frac{1}{2}\pi G(x)\right)-\frac{\pi^{2}(-k-1)g{\left(x\right)}^{2}\sec^{4}\left(\frac{1}{2}\pi G(x)\right)}{4s^{2}{\left(\frac{\tan\left(\frac{1}{2}\pi G(x)\right)}{s}+1\right)}^{2}}+\frac{\pi^{2}(-k-1)g{\left(x\right)}^{2}\tan\left(\frac{1}{2}\pi G(x)\right)\sec^{2}\left(\frac{1}{2}\pi G(x)\right)}{2s\left(\frac{\tan\left(\frac{1}{2}\pi G(x)\right)}{s}+1\right)}+\frac{1}{2}\pi^{2}g{\left(x\right)}^{2}\sec^{2}\left(\frac{1}{2}\pi G(x)\right).$$

### 4.4 Hazard function's shapes

This Eq. produces the hrf's h(x) critical points.

(7) $$\frac{d\;\log\;h(x)}{dx}=\frac{g^{\prime }(x)}{g(x)}-\frac{\pi g(x)\sec^{2}\left(\frac{1}{2}\pi G(x)\right)}{2s\left(\frac{\tan\left(\frac{1}{2}\pi G(x)\right)}{s}+1\right)}+\pi g(x)\tan\left(\frac{1}{2}\pi G(x)\right),$$

and there may be several roots to (7). Let $\lambda (x)=d^{2}\;\log[h(x)]/d\;x^{2}$, so we can say that

$$\lambda (x)=\frac{g^{\prime \prime }(x)}{g(x)}-\frac{g^{\prime }{\left(x\right)}^{2}}{g{\left(x\right)}^{2}}-\frac{\pi g^{\prime }(x)\sec^{2}\left(\frac{1}{2}\pi G(x)\right)}{2s\left(\frac{\tan\left(\frac{1}{2}\pi G(x)\right)}{s}+1\right)}+\frac{1}{2}\pi^{2}g{\left(x\right)}^{2}\sec^{2}\left(\frac{1}{2}\pi G(x)\right)+\pi g^{\prime }(x)\tan\left(\frac{1}{2}\pi G(x)\right)+\frac{\pi^{2}g{\left(x\right)}^{2}\sec^{4}\left(\frac{1}{2}\pi G(x)\right)}{4s^{2}{\left(\frac{\tan\left(\frac{1}{2}\pi G(x)\right)}{s}+1\right)}^{2}}-\frac{\pi^{2}g{\left(x\right)}^{2}\tan\left(\frac{1}{2}\pi G(x)\right)\sec^{2}\left(\frac{1}{2}\pi G(x)\right)}{2s\left(\frac{\tan\left(\frac{1}{2}\pi G(x)\right)}{s}+1\right)}.$$

### 4.5 Moments, skewness, kurtosis and generating function

Suppose if Y is a random variable, and use G(x) as the baseline for your analysis. We can calculate the moments by the method described by Greenwood et al. [28] in this formula

$$\tau_{r,k}=\text{E}[Y^{r}\;G{\left(Y\right)}^{k}]=\int_{-\infty }^{\infty }x^{r}\;G{\left(x\right)}^{k}\;g(x)dx.$$

We may write (5) from the equation.

$$\mu_{r}^{\prime }=\text{E}(X^{r})=\sum_{i=1}^{\infty }\;\sum_{j=0}^{\infty }\;w_{i,j}\;h_{\left(2j+i\right)}(x)\tau_{r,(2j+i)},$$

where $\tau_{r,(2j+i)}=\int_{0}^{1}Q_{G}{\left(u\right)}^{r}\;u^{\left(2j+i\right)}du$ may be calculated numerically from any baseline qf.

**Figure 5:**
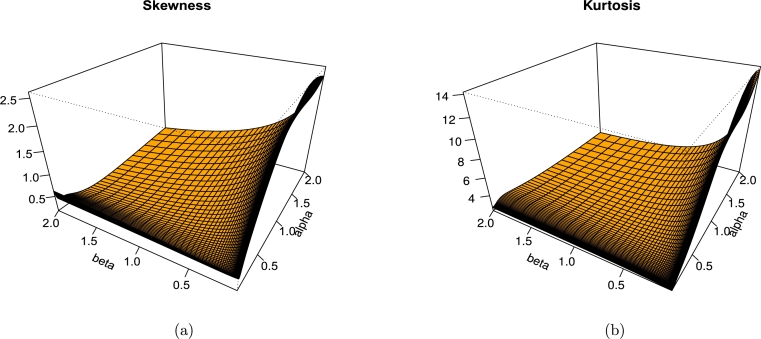
LT-W's plots regarding (a) skewness (b) kurtosis.

Calculations may be made to determine the skewness and kurtosis of the LT-W distribution using

$$Skewness(X)=\frac{\overset{´}{\mu_{3}}-3\;\overset{´}{\mu_{2}}\;\overset{´}{\mu_{1}}+2\;{\overset{´}{\mu_{1}}}^{3}}{{\left(\overset{´}{\mu_{2}}-{\overset{´}{\mu_{1}}}^{2}\right)}^{\frac{3}{2}}},$$

and

$$Kurtosis(X)=\frac{\overset{´}{\mu_{4}}-4\;\overset{´}{\mu_{1}}\;\overset{´}{\mu_{3}}+6\;{\overset{´}{\mu_{1}}}^{2}\;\overset{´}{\mu_{3}-3\;{\overset{´}{\mu_{1}}}^{4}}}{\overset{´}{\mu_{2}}-{\overset{´}{\mu_{1}}}^{2}},$$

the graph in Fig. 5 shows the LT-W's plots regarding Fig. 5 (a) skewness, and Fig. 5 (b) kurtosis.

For your convenience, we have provided two distinct formulas that can be used to determine the mgf. $M(t)=E(e^{t\;X})$ of *X*. Eq. (5) is where the initial formula for M(t) is generated as follows:

(8) $$M(t)=\sum_{i=1}^{\infty }\;\sum_{j=0}^{\infty }\;w_{i,j}\;M_{\left(2j+i\right)}(t),$$

such that $M_{\left(2j+i\right)}(t)$ is the exp-G generating function, and (2j+i) is the power parameter. Eq. (8) may alternatively be written as

$$M(t)=\sum_{i=1}^{\infty }\;\sum_{j=0}^{\infty }\;(2j+i)\;\;w_{i,j}\;\rho_{\left(2j+i\right)}(t),$$

where this quantity $\rho_{2j+i}(t)=\int_{0}^{1}\exp\left[s\;Q_{G}(u)\right]u^{\left(2j+i\right)}du$ may be calculated numerically.

### 4.6 Order statistics

Suppose we have generated samples from the LT-G distribution family. $X_{i:n}$ density function, may be written as

$$f_{i:n}(x)=K\;\sum_{p=0}^{n-i}{\left(-1\right)}^{p}\;\left(\begin{array}{c} n-i\\ p \end{array}\right)\;f(x)\;F{\left(x\right)}^{p+i-1},$$

where R=n!/[(i−1)!(n−i)!]. We may express the density function of $X_{i:n}$ as follows similar algebraic advancements of Nadarajah et al. [45]

(9) $$f_{i:n}(x)=\sum_{m,r,t=0}^{\infty }\;d_{m,r,t}\;h_{\left(2(r+t)+m\right)}(x),$$

here $h_{\left(2(r+t)+m\right)}(x)$ shows the exp-G density possessing power parameter (2(r+t)+m).

$$d_{m,r,t}=\sum_{j,l=0}^{\infty }\;R\;\frac{{\left(-1\right)}^{j+l}}{s^{m+1}}\left(\begin{array}{c} n-i\\ j \end{array}\right)\;\left(\begin{array}{c} i+j-1\\ l \end{array}\right)\;\left(\begin{array}{c} -k(l+1)-1\\ m \end{array}\right)b_{r}(m)\;c_{t}(2){\left[\frac{\pi }{2}\right]}^{2(r+t)+m+1}\;\frac{1}{2(r+t)+m+1},$$

where $b_{r}(m)$ and $c_{t}(2)$ are constants. This section's major conclusion is an Eq. (9).

## 5 Estimation of parameters

Here, we advise on parameter estimates utilizing the maximum likelihood technique (MLE).

### 5.1 Maximum likelihood estimation

We exclusively use the MLE approach to estimate the values of the family's unknown parameters using complete samples. Let $\Theta ={\left(s\;,k\;,\xi \right)}^{⊤}$ describe the parameter's vector. We can obtain the log-likelihood function for Θ can indeed be phrased in the following way:

$$\ell_{n}(\Theta )=n\log(k)+n\log(\pi )+\sum_{i=1}^{n}\log(g(x_{i};\xi ))-(k+1)\sum_{i=1}^{n}\log\left(\frac{\tan\left(\frac{1}{2}\;\pi G(x_{i};\xi )\right)}{s}+1\right)+\sum_{i=1}^{n}\log\left(\sec^{2}\left(\frac{1}{2}\pi G(x_{i};\xi )\right)\right)-n\log(s)-n\log(2).$$

We can say that $U_{n}(\Theta )={\left(\partial \ell_{n}/\partial s,\partial \ell_{n}/\partial k,\partial \ell_{n}/\partial \xi \right)}^{⊤}$ are

$$\frac{\partial \ell_{n}}{\partial s}=-\frac{n}{s}+\frac{n\;s\;(-k-1)\;\left(\frac{1}{s}-\frac{\tan\left(\frac{1}{2}\pi \;G(x)\right)+s}{s^{2}}\right)}{\tan\left(\frac{1}{2}\pi^{2}G(x)\right)+s},$$

$$\frac{\partial \ell_{n}}{\partial k}=\frac{n}{k}-n\log\left(\frac{\tan\left(\frac{1}{2}\pi \;G(x)\right)+s}{s}\right),$$

$$\frac{\partial \ell_{n}}{\partial \xi }=\sum_{i=1}^{n}\frac{g{\left(x_{i};\xi \right)}^{\left(\xi \right)}}{g(x_{i};\xi )}+\pi \;\sum_{i=1}^{n}G{\left(x_{i};\xi \right)}^{\left(\xi \right)}\;\tan\left(\frac{\pi }{2}\;G(x_{i};\xi )\right)+\frac{\pi }{2}\;(k+1)\;\sum_{i=1}^{\infty }\;G{\left(x_{i}\xi \right)}^{\left(\xi \right)}\frac{\left(\frac{\sec^{2}\left(\frac{1}{2}\pi G(x_{i};\xi )\right)}{s}\right)}{1+\left(\frac{\tan\left(\frac{1}{2}\pi \;G(x_{i};\xi )\right)}{s}\right)}.$$

Such that $g^{\left(\xi \right)}(\cdot )$ denotes the function *g*'s derivative with regard to *ξ*.

## 6 Simulation study

**Figure 6:**
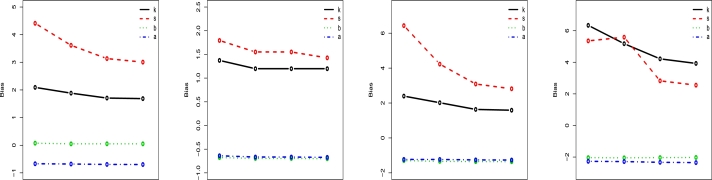
LT-W's B plots on distinct parametric values.

**Figure 7:**
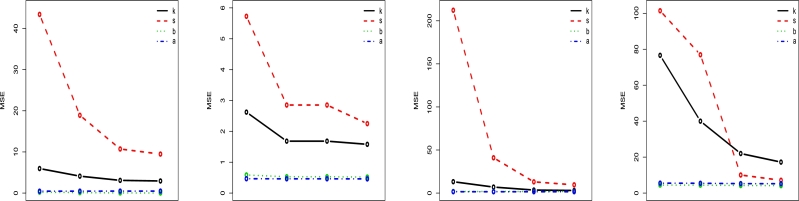
LT-W's Mean square error (M) plots on distinct parametric values.

**Figure 8:**
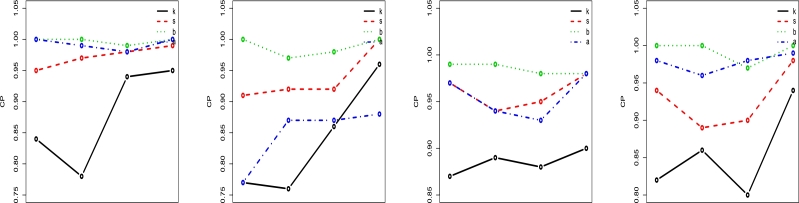
LT-W's Coverage probability (CP) plots on distinct parametric values.

Simulation research is done to find out how well the MLEs of the LT-W parameters work. We run 1000 samples of the LT-W model with n=50, 100, 300, and 500 for different parametric variables. Estimates are calculated based on the Bias (B), mean square error (M), Coverage Probability (C), and Average Width (AvW). The examination was carried out using R-software, and the results are shown in Table 3, Table 4 and Figure 6, Figure 7, Figure 8. The numbers in Table 3, Table 4 and The graphs that are shown in Figure 6, Figure 7, Figure 8 demonstrate that the estimates may be relied upon totally and, more significantly, also that estimates are not too far off from the actual numbers when considering these sample sizes. Table 3, Table 4 and Figure 6, Figure 7, Figure 8 demonstrate that the Biases, MSEs drop while CP increases as n grows. We conclude from this simulation analysis that the maximum likelihood technique is useful for calculating the LT-W parameters. As n becomes larger, the estimates of the parameter values have a greater tendency to approach the true values. This demonstrates that the asymptotic normal distribution may serve as a reasonable approximation for the distribution of the MLEs across finite samples. It is possible to improve the normal approximation by making B adjustments to these estimators. Basic models can be used to approximate their biases analytically.

**Table 3:** Estimated B, M, coverage probability and average width of MLE of parameters with sample size = 50, 100, 300, 500.

**Table:** MLE(k=0.2,s=0.5,b=0.8,a=1.2) *n* B M C AvW 50 *k* 2.092 5.967 0.84 18.965 *s* 4.411 43.417 0.95 2.109 *b* 0.074 0.222 1.00 5.117 *a* -0.670 0.464 1.00 .089 100 *k* 1.884 4.117 0.78 8.965 *s* 3.614 18.905 0.97 1.443 *b* 0.049 0.117 1.00 3.250 *a* -0.680 0.472 0.99 2.145 300 *k* 1.706 3.093 0.94 3.681 *s* 3.134 10.717 0.98 0.825 *b* 0.051 0.043 0.99 1.701 *a* -0.695 0.487 0.98 1.355 500 *k* 1.686 2.946 0.95 2.641 *s* 3.006 9.481 0.99 0.638 *b* 0.050 0.027 1.00 1.297 *a* -0.698 0.490 1.00 1.150	**Table:** MLE(k=0.8,s=1.2,b=1.5a=1.8) *n* B M C AvW 50 *k* 1.373 2.623 0.77 5.860 *s* 1.794 5.728 0.91 1.463 *b* -0.677 0.587 1.00 3.376 *a* -0.639 0.465 0.77 2.849 100 *k* 1.198 1.683 0.76 2.620 *s* 1.551 2.853 0.92 0.820 *b* -0.696 0.529 0.97 1.773 *a* -0.666 0.463 0.87 2.021 300 *k* 1.198 1.683 0.86 2.620 *s* 1.551 2.853 0.92 0.820 *b* -0.696 0.529 0.98 1.773 *a* -0.666 0.463 0.87 2.021 500 *k* 1.198 1.580 0.96 1.855 *s* 1.427 2.250 0.99 0.630 *b* -0.701 0.519 1.00 1.361 *a* -0.673 0.460 0.88 1.807

**Table 4:** Estimated B, M, coverage probability and average width of MLE of parameters sample size = 50, 100, 300, 500.

**Table:** MLE(k=1.5,s=1.8,b=2.1,a=2.6) *n* B M C AvW 50 *k* 2.397 13.236 0.87 53.562 *s* 6.433 212.256 0.97 2.116 *b* -1.297 1.883 0.99 9.850 *a* -1.230 1.600 0.97 6.295 100 *k* 2.018 6.999 0.89 20.006 *s* 4.229 41.003 0.94 1.451 *b* -1.341 1.912 0.99 6.307 *a* -1.232 1.572 0.94 4.522 300 *k* 1.634 3.536 0.88 7.405 *s* 3.09 13.052 0.95 0.827 *b* -1.354 1.879 0.98 3.237 *a* -1.258 1.606 0.93 2.961 500 *k* 1.588 3.048 0.90 5.072 *s* 2.817 9.568 0.98 0.636 *b* -1.360 1.875 0.99 2.464 *a* -1.265 1.615 0.98 2.567	**Table:** MLE(k=1.1,s=0.7,b=3.0,a=3.5) *n* B M C AvW 50 *k* 6.339 76.611 0.82 39.464 *s* 5.356 101.401 0.94 2.794 *b* -2.036 4.473 1.00 20.842 *a* -2.270 5.532 0.98 11.650 100 *k* 5.178 40.003 0.86 34.058 *s* 5.589 76.934 0.89 1.939 *b* -2.049 4.428 1.00 12.680 *a* -2.285 5.459 0.96 7.555 300 *k* 4.219 22.010 0.80 5.290 *s* 2.826 10.056 0.90 1.123 *b* -2.034 4.219 0.97 6.184 *a* -2.332 5.270 0.98 4.259 500 *k* 3.929 17.232 0.94 3.430 *s* 2.553 7.134 0.98 0.869 *b* -2.025 4.142 1.00 4.553 *a* -2.350 5.222 0.99 3.427

## 7 Applications and data analysis

The LT-W distribution's usefulness is illustrated in this part by two applications to absolute life data sets, along with empirical proof that it can be used instead of a few well-known models.

### 7.1 Goodness-of-fit statistics

Certain high-quality goodness-of-fit measures, such as the log-likelihood ($\overset{ˆ}{\ell }$) function, were measured at the MLEs to compare the models, Akaike information criterion (AIC), Anderson-Darling ($A^{⁎}$), Cram'er—von Mises ($W^{⁎}$) and Kolmogrov-Smirnov (K-S) are computed. The statistics $A^{⁎}$ and $W^{⁎}$ are detailed in Chen et al. [18]. The model with the lowest values for these statistics may be chosen as the best fit for the data. The R-software is used to do the required computations.

Table 5 includes the MLEs and their accompanying standard errors (in parentheses) of the model parameters, while Table 6 lists the statistics AIC, $A^{⁎}$, $W^{⁎}$ and K-S. Table 6 shows that, when compared to other models, LT-W gives a better match to both data sets.

**Table 5:** MLEs of the LT-W and fitted models using the Wheaton river data along with the corresponding SEs (given in parentheses).

Model	*k*	*s*	*b*	*a*	p-value	K-S	
LT-W	2.9651	3.7781	0.8301	13.4326	0.9818	0.0548	
	(0.0287)	(0.0347)	(0.0213)	(0.0213)			

LW	0.1449	0.03124	1.6396	6.3766	0.9651	0.0587	
	(0.0471)	(0.0378)	(0.2819)	(2.1639)			

KW	0.5312	1.3170	0.0105	1.4030	0.4034	0.1051	
	(0.2097)	(1.1531)	(0.0135)	(0.3335)			

TLW	0.5858	0.0124	1.2712	0.06	0.17		
	(0.2198)	(0.0155)	(0.3199)				

BXIIW	0.2098	0.2266	4.0058	56.6702	0.4017	0.1053	
	(3.6830)	(2.5421)	(44.9236)	(126.0379)			

BW	0.5513	0.4971	0.0494	1.2934	0.3772	0.1074	
	(0.2798)	(0.6605)	(0.1281)	(0.4598)

**Table 6:** The results for fitness measures for the first data.

Model	$-2\overset{ˆ}{\ell }$	*AIC*	*BIC*	*HQIC*	*CAIC*	*W*^⁎^	*A*^⁎^
LT-W	247.12	502.24	511.35	505.87	502.84	0.027	0.192
LW	247.49	503	512.09	506.60	503.58	0.031	0.210
KW	250.95	509.91	519.02	513.54	510.51	0.106	0.645
TLW	251.04	508.09	514.92	510.81	508.44	0.110	0.663
BXIIW	251.59	511.19	520.30	514.81	511.79	0.141	0.801
BW	250.97	509.95	519.06	513.58	510.55	0.103	0.633

### 7.2 Application 1 on Wheaton river data

The LT-W distribution's influence is dressed up with a real data set. The first data set from Choulakian and Stephens [23]. The data are: 1.7, 2.2, 14.4, 1.1, 0.4, 20.6, 5.3, 0.7, 1.9, 13.0, 12.0, 9.3, 1.4, 18.7, 8.5, 25.5, 11.6, 14.1, 22.1, 1.1, 2.5, 14.4, 1.7, 37.6, 0.6, 2.2, 39.0, 0.3, 15.0, 11.0, 7.3, 22.9, 1.7, 0.1, 1.1, 0.6, 9.0, 1.7, 7.0, 20.1, 0.4, 2.8, 14.1, 9.9, 10.4, 10.7, 30.0, 3.6, 5.6, 30.8, 13.3, 4.2, 25.5, 3.4, 11.9, 21.5, 27.6, 36.4, 2.7, 64.0, 1.5, 2.5, 27.4, 1.0, 27.1, 20.2, 16.8, 5.3, 9.7, 27.5, 2.5, 27.0.

It is a right-skewed data set with a big right tail (skewness = 1.5 and kurtosis = 3.19).

#### 7.2.1 Outcomes of application 1

The values of the MLEs and their accompanying standard errors (in parentheses) of the model parameters are listed in Table 5. The LT-W model is compared to the Lomax Weibull (LW), Kumaraswamy Weibull (KW), transmuted Lomax Weibull (TLW), Burr XII-Inverse Weibull (BurrXIIW), and beta Weibull (BW) distributions in Table 6 among all fitted models, the KwT-W model has the lowest values for the AIC, BIC, CAIC, HQIC, $W^{⁎}$, $A^{⁎}$, p-value and k-s value statistics.

**Figure 9:**
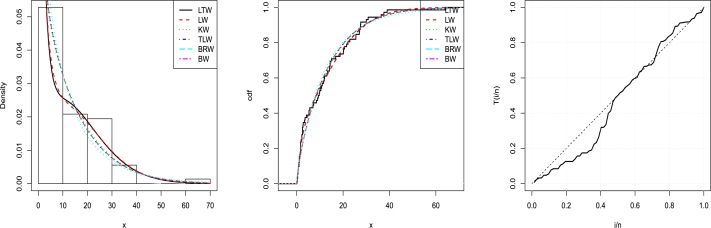
Graphs for estimated pdfs (Left), estimated cdfs (Middle) and TTT plot (Right) of LT-W distribution using Wheaton river data.

As a result, the KwT-W model may be selected as the best model to describe the existing data. Fig. 9 plots support the fitted KwT-W distribution more than the other nested and non-nested models.

### 7.3 Application 2 on strengths of 1.5 cm glass fibers data

The significance of the LT-W distribution is explained in depth using an actual data set. It is comprised of 63 observations of the strengths of glass fibers that are 1.5 cm in length, see Smith and Naylor, [55]). The information is as follows: 0.55, 0.93, 1.25, 1.36, 1.49, 1.52, 1.58, 1.61, 1.64, 1.68, 1.73, 1.81, 2.00, 0.74, 1.04, 1.27, 1.39, 1.49, 1.53, 1.59, 1.61, 1.66, 1.68, 1.76, 1.82, 2.01, 0.77, 1.11, 1.28, 1.42, 1.50, 1.54, 1.60, 1.62, 1.66, 1.69, 1.76, 1.84, 2.24, 0.81, 1.13, 1.29, 1.48, 1.50, 1.55, 1.61, 1.62, 1.66, 1.70, 1.77, 1.84, 0.84, 1.24, 1.30, 1.48, 1.51, 1.55, 1.61, 1.63, 1.67, 1.70, 1.78, 1.89.

### 7.4 Outcomes of application 2

A comparison is made between the LT-W model and the WTBXII, WBXII, KwEBXII, BBXII, WL, Wfr, and BXII distributions in Table 7, Table 8. Among all fitted models, the WT-BXII model has the lowest values for the AIC, BIC, CAIC, HQIC, $W^{⁎}$, $A^{⁎}$, p-value and k-s value statistics.

**Table 7:** MLEs of LT-W and fitted models to data set 2, the corresponding SEs (given in parentheses).

Model	*k*	*s*	*b*	*a*	p-value	K-S	
LT-W	0.7557	.5220	3.8308	1.1863	0.6861	0.0900	
	(0.4257)	(7.8093)	(1.0551)	(0.1465)			

WT-BXII	0.0551	0.5438	7.8425	1.4846	0.2469	0.12879	
	(0.0223)	(0.4500)	(5.7808)	(0.5877)			

WL	0.0151	3.3958	7.0485	6.9662	0.1612	0.1413	
	(0.0211)	(0.9310)	(12.0152)	(12.8202)			

WFr	0.0133	4.2311	0.5722	1.1316	0.06	0.17	
	(0.0171)	(3.1847)	(0.1724)	(0.9469)			

BBXII	103.5185	174.5075	0.5598	0.5776	0.0023	0.2317	
	(245.8979)	(401.0985)	(0.6915)	(1.1619)			

WBXII	0.0121	2.9850	1.5763	1.4631	0.11	0.15	
	(0.0121)	(2.607)	(1.028)	(0.599)			

	*α*	*β*	*a*	*b*	*c*	p-value	K-S
KEBXII	4.2737	658.3955	0.8542	1.3141	4.2649	0.0281	0.1839
	(36.7061)	(745.2032)	(0.6580)	(1.4898)	(36.6305)

**Table 8:** The results for fitness measures for the second data.

Model	$-2\overset{ˆ}{\ell }$	*AIC*	*BIC*	*HQIC*	*CAIC*	*W*^⁎^	*A*^⁎^
LT-W	11.01	30.02	38.59	33.39	30.71	0.0754	0.4334
WT-BXII	13.167	34.33	42.90	37.70	35.02	0.1460	0.8098
WBXII	14.80	37.59	46.16	40.96	38.28	0.2176	1.1995
WL	14.42	36.85	45.42	40.22	37.54	0.1879	1.0467
WFr	16.11	40.23	48.81	43.60	40.92	0.2818	1.5420
KwEBXII	18.29	46.59	57.31	50.18	47.65	0.3675	2.0112
BBXII	28.07	64.15	72.72	67.52	64.84	0.703	3.8426

**Figure 10:**
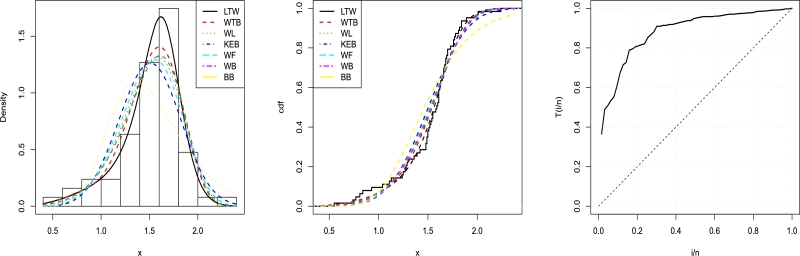
Graphs for estimated pdfs (Left), estimated cdfs (Middle), and TTT plot (Right) of LT-W distribution using strengths of 1.5 cm glass fiber data.

As a consequence of this, the LT-W model may be chosen as the model that is best suited to describe the data that is currently available. The plots in Fig. 10 provide stronger evidence in favor of the fitted LT-W distribution than any of the other models.

## 8 Final observations

In essence, this study presents a novel family of Lomax tangent-G distributions with forms for the hazard function that includes growing, decreasing, bathtub, and many other variations. We look into some of its structural features, including density and hazard function forms. To find the values of the parameters during the estimation using a Monte Carlo simulation, we used a traditional method of estimation. Through the use of goodness-of-fit metrics, we demonstrated the superiority of the new distribution. We come to the conclusion that, in terms of match quality, the proposed model consistently outperforms alternatives. The proposed family and model are expected to be applied more frequently in the life sciences.

## 9 Future work

In the future, we will be able to apply this distribution to different kinds of data, be it medical or engineering data. Also, we may apply bi-variate distribution to this work as [25], [26], [27], [42] and [9]. We suggest using the ranked set sample method in the future like [49], [8], and [50]. Also, we can use different estimation methods to determine proposed model estimators like [2], [57], [58]. The proposed model can be used for analyzing COVID-19 real data sets like [4], [13], [31], [32], [38], [41], [48].

## CRediT authorship contribution statement

**Sajid Mehboob Zaidi:** Formal analysis, Data curation, Conceptualization. **Zafar Mahmood:** Funding acquisition, Conceptualization. **Mintodê Nicodème Atchadé:** Methodology, Formal analysis. **Yusra A. Tashkandy:** Formal analysis, Conceptualization. **M.E. Bakr:** Investigation, Funding acquisition. **Ehab M. Almetwally:** Methodology, Formal analysis, Data curation, Conceptualization. **Eslam Hussam:** Methodology, Funding acquisition, Formal analysis, Conceptualization. **Ahmed M. Gemeay:** Investigation. **Anoop Kumar:** Formal analysis.

## Declaration of Competing Interest

The authors declare no conflict of interest.

## Data Availability

The data supporting this study's findings are available within the article.

## References

[1] Al-Babtain A.A., Elbatal I., Chesneau C., Elgarhy M. (2020). Sine Topp-Leone-G family of distributions: theory and applications. Open Phys..

[2] Al-Babtain A.A., Gemeay A.M., Afify A.Z. (2021). Estimation methods for the discrete Poisson Lindley and discrete Lindley distributions with actuarial measures and applications in medicine. J. King Saud Univ., Sci..

[3] Al-Babtain Abdulhakim A., Elbatal Ibrahim, Al-Mofleh Hazem, Gemeay Ahmed M., Afify Ahmed Z., Sarg Abdullah M. (2021). The flexible Burr X-G family: properties, inference, and applications in engineering science. Symmetry.

[4] Aldallal R., Gemeay A.M., Hussam E., Kilai M. (2022). Statistical modeling for COVID 19 infected patient's data in Kingdom of Saudi Arabia. PLoS ONE.

[5] Ali A. (2020). Sine power Lomax model with application to bladder cancer data. Nanosci. Nanotechnol. Lett..

[6] Alizadeh M.A., Korkmaz MÇ, Almamy J.A., Ahmed A.AE. (2021). Another odd log-logistic logarithmic class of continuous distributions. J. Stat. Stat. Actuar. Sci..

[7] Aljarrah M.A., Lee C., Famoye F. (2014). A method of generating T-X family of distributions using quantile functions. J. Stat. Distrib. Appl..

[8] Aljohani H.M., Almetwally E.M., Alghamdi A.S., Hafez E.H. (2021). Ranked set sampling with application of modified Kies exponential distribution. Alex. Eng. J..

[9] Almetwally E.M., Muhammed H.Z., El-Sherpieny E.S.A. (2020). Bivariate Weibull distribution: properties and different methods of estimation. Ann. Data Sci..

[10] Almetwally Ehab M. (2022). The odd Weibull inverse Topp–Leone distribution with applications to COVID-19 data. Ann. Data Sci..

[11] Almetwally Ehab M., Sabry Mohamed A.H., Alharbi Randa, Alnagar Dalia, Mubarak Sh.A.M., Hafez E.h. (2021). Marshall–Olkin alpha power Weibull distribution: different methods of estimation based on type-I and type-II censoring. Complexity.

[12] Almongy Hisham M., Almetwally Ehab M., Aljohani Hassan M., Alghamdid Abdulaziz S., Hafeze E.H. (2021). A new extended Rayleigh distribution with applications of COVID-19 data. Results Phys..

[13] Almuqrin M.A., Gemeay A.M., Abd El-Raouf M.M., Kilai M., Aldallal R., Hussam E. (2022). A flexible extension of reduced Kies distribution: properties, inference, and applications in biology. Complexity.

[14] Altun E., Korkmaz M.C., El-Morshedy M., Eliwa M.S. (2021). A new flexible family of continuous distributions: the additive odd-G family. Mathematics.

[15] Alzaatreh A., Lee C., Famoye F. (2013). A new method for generating families of continuous distributions. Metron.

[16] Bhatti F.A., Korkmaz M.C., Hamedani G.G., Cordeiro G.M., Ahmad M. (2019). On Burr III Marshal Olkin family: development, properties, characterizations and applications. J. Stat. Distrib. Appl..

[17] Chakraborty S., Hazarika P.J., Ali M.M. (2012). A new skew logistic distribution and its properties. Pak. J. Stat..

[18] Chen G., Balakrishnan N. (1995). A general purpose approximate goodness-of-fit test. J. Qual. Technol..

[19] Chen P., Ye Z.S. (2017). Approximate statistical limits for a gamma distribution. J. Qual. Technol..

[20] Chen Piao, Ye Zhi-Sheng (2017). Estimation of field reliability based on aggregate lifetime data. Technometrics.

[21] Chesneau C., Jamal F. (2020). The sine Kumaraswamy-G family of distributions. J. Math. Ext..

[22] Chesneau C., Bakouch H.S., Hussain T. (2019). A new class of probability distributions via cosine and sine functions with applications. Commun. Stat., Simul. Comput..

[23] Choulakian V., Stephens M.A. (2001). Goodness-of-fit tests for the generalized Pareto distribution. Technometrics.

[24] Cordeiro M., Emrah A., Korkmaz M., Haitham M. (2020). The XGamma family: censored regression modelling and applications. REVSTAT Stat. J..

[25] El-Sherpieny E.S.A., Almetwally E.M., Muhammed H.Z. (2022). Bivariate Weibull-G family based on copula function: properties, Bayesian and non-Bayesian estimation and applications. Stat. Optim. Inf. Comput..

[26] El-Sherpieny E.S.A., Muhammed H.Z., Almetwally E.M. (2022). Accelerated life testing for bivariate distributions based on progressive censored samples with random removal. J. Stat. Appl. Probab..

[27] El-Sherpieny E.S.A., Muhammed H.Z., Almetwally E.M. (2022). Progressive type-II censored samples for bivariate Weibull distribution with economic and medical applications. Ann. Data Sci..

[28] Greenwood J.A., Landwehr J.M., Matalas N.C. (1979). Probability weighted moments: definition and relation to parameters of several distributions expressable in inverse form. Water Resour. Res..

[29] Hamedani G.G., Korkmaz MÇ, Butt N.S., Yousof H.M. (2021). The type I quasi Lambert family: properties, characterizations and different estimation methods. Pak. J. Stat. Oper. Res..

[30] Hassan Amal S., Almetwally Ehab M., Ibrahim Gamal M. (2021). Kumaraswamy inverted Topp–Leone distribution with applications to COVID-19 data. Comput. Mater. Continua.

[31] Hossam E., Abdulrahman A.T., Gemeay A.M., Alshammari N., Alshawarbeh E., Mashaqbah N.K. (2022). A novel extension of Gumbel distribution: statistical inference with Covid-19 application. Alex. Eng. J..

[32] Kamal M., Atchadé M.N., Sokadjo Y.M., Siddiqui S.A., Riad F.H., Abd El-Raouf M.M., Aldallal R., Hussam E., Alshanbari H.M., Alsuhabi H., Gemeay A.M. (2023). Influence of COVID-19 vaccination on the dynamics of new infected cases in the world. Math. Biosci. Eng..

[33] Kharazmi O., Alizadeh M., Contreras-Reyes Javier E., Haghbin H. (2022). Arctan-based family of distributions: properties, survival regression, Bayesian analysis and applications. Axioms.

[34] Kharazmi Omid, Saadatinik Ali, Tamandi Mostafa (2015). Hyperbolic sine - Weibull distribution and its applications. Int. J. Math. Comput..

[35] Korkamaz M.C., Yousof H.M., Alizadeh M., Hamdani G.G. (2019). The Topp-Leone generalized odd log-logistic family of distributions: properties, characterizations and applications. Commun. Fac. Sci. Univ. Ank. Sér. A1 Math. Stat..

[36] Korkmaz M.Ç., Genç A.I. (2017). A new generalized two-sided class of distributions with an emphasis on two-sided generalized normal distribution. Commun. Stat., Simul. Comput..

[37] Korkmaz M.C., Altun E., Yousof H.M., Hamdani G.G. (2020). The Hjorth's IDB generator of distributions: properties, characterizations, regression modeling and applications. J. Stat. Theory Appl..

[38] Liu X., Ahmad Z., Gemeay A.M., Abdulrahman A.T., Hafez E.H., Khalil N. (2021). Modeling the survival times of the COVID-19 patients with a new statistical model: a case study from China. PLoS ONE.

[39] Luo Chunling, Shen Lijuan, Xu Ancha (2022). Modelling and estimation of system reliability under dynamic operating environments and lifetime ordering constraints. Reliab. Eng. Syst. Saf..

[40] Mahmood Z., Chesneau C., Tahir M.H. (2019). A new sine-G family of distributions: properties and applications. Bull. Comput. Appl. Math..

[41] Meriem B., Gemeay A.M., Almetwally E.M., Halim Z., Alshawarbeh E., Abdulrahman A.T., Abd El-Raouf M.M., Hussam E. (2022). The power XLindley distribution: statistical inference, fuzzy reliability, and COVID-19 application. J. Funct. Spaces.

[42] Muhammed H.Z., El-Sherpieny E.S.A., Almetwally E.M. (2021). Dependency measures for new bivariate models based on copula function. Inf. Sci. Lett..

[43] Muse A.H., Tolba A.H., Fayad E., Ali O.A.A., Nagy M., Yusuf M. (2021). Modelling the COVID-19 mortality rate with a new versatile modification of the log-logistic distribution. Comput. Intell. Neurosci..

[44] Nadarajah S., Kotz S. (2006). Beta trigonometric distribution. Port. Econ. J..

[45] Nadarajah S., Cordeiro G.M., Ortega E.M.M. (2015). The Zografos-Balakrishnan–G family of distributions: mathematical properties and applications. Commun. Stat., Theory Methods.

[46] Nagarjuna V.B.V., Vardhan R.V., Chesneau C. (2020). On the accuracy of the sine power Lomax model for data fitting. Modelling.

[47] Raab D.H., Green E.H. (1961). A cosine approximation to the normal distribution. Psychometrika.

[48] Riad F.H., Alruwaili B., Gemeay A.M., Hussam E. (2022). Statistical modeling for COVID 19 virus spread in Kingdom of Saudi Arabia and Netherlands. Alex. Eng. J..

[49] Sabry M.A., Almetwally E.M., Almongy H.M., Ibrahim G.M. (2021). Assessing the performance of some ranked set sampling designs using hybrid approach. Comput. Mater. Continua.

[50] Sabry M.H., Almetwally E.M. (2021). Estimation of the exponential Pareto distribution's parameters under ranked and double ranked set sampling designs. Pak. J. Stat. Oper. Res..

[51] Salahuddin N., Khalil A., Mashwani W.K., Shah H., Jomsri P., Panityakul T. (2021). Meta-Heuristic Techniques for Solving Computational Engineering Problems.

[52] Shi Gang, Zhuang Rujun, Sheng Yuhong (2021). Sine entropy of uncertain random variables. Symmetry.

[53] Shrahili M., Elbatal I., Almutiry W., Elgarhy M. (2020). Estimation of sine inverse exponential model under censored schemes. J. Math..

[54] Shrahili M., Elbatal I., Elgarhy M. (2020). Sine half-logistic inverse Rayleigh distribution: properties, estimation, and applications in biomedical data. J. Math..

[55] Smith Richard L., Naylor J.C. (1987). A comparison of maximum likelihood and Bayesian estimators for the three-parameter Weibull distribution. J. R. Stat. Soc., Ser. C, Appl. Stat..

[56] Souza L. (2015).

[57] Teamah A.E.A., Elbanna A.A., Gemeay A.M. (2020). Right truncated Fréchet-Weibull distribution: statistical properties and application. Delta J. Sci..

[58] Teamah A.E.A., Elbanna A.A., Gemeay A.M. (2021). Heavy-tailed log-logistic distribution: properties, risk measures and applications. Stat. Optim. Inf. Comput..

[59] Zhuang Liangliang, Xu Ancha, Wang Xiao-Lin (2023). A prognostic driven predictive maintenance framework based on Bayesian deep learning. Reliab. Eng. Syst. Saf..

